# Intrinsic elasticity of nucleosomes is encoded by histone variants and calibrated by their binding partners

**DOI:** 10.1073/pnas.1911880116

**Published:** 2019-11-11

**Authors:** Daniël P. Melters, Mary Pitman, Tatini Rakshit, Emilios K. Dimitriadis, Minh Bui, Garegin A. Papoian, Yamini Dalal

**Affiliations:** ^a^Laboratory Receptor Biology and Gene Expression, Center for Cancer Research, National Cancer Institute, Bethesda, MD 20892;; ^b^Department of Chemistry and Biochemistry, Institute for Physical Science and Technology, University of Maryland, College Park, MD 20742;; ^c^Scanning Probe Microscopy Unit, Biomedical Engineering and Physical Science Shared Resource, National Institute for Biomedical Imaging and Bioengineering, National Institutes of Health, Bethesda, MD 20892

**Keywords:** chromatin, histone variants, elasticity, computational modeling, epigenetics

## Abstract

Histone variants fine-tune transcription, replication, DNA damage repair, and faithful chromosome segregation. Whether and how nucleosome variants encode unique mechanical properties to their cognate chromatin structures remains elusive. Here, using in silico and in vitro nanoindentation methods, extending to in vivo dissections, we report that histone variant nucleosomes are intrinsically more elastic than their canonical counterparts. Furthermore, binding proteins, which discriminate between histone variant nucleosomes, suppress this innate elasticity and also compact chromatin. Interestingly, when we overexpress the binding proteins in vivo, we also observe increased compaction of chromatin enriched for histone variant nucleosomes, correlating with diminished access. Taken together, these data suggest a plausible link between innate mechanical properties possessed by histone variant nucleosomes, the adaptability of chromatin states in vivo, and the epigenetic plasticity of the underlying locus.

## Significance

Nucleosomes are the base units that organize eukaryotic genomes. Besides the canonical histone, histone variants create unique local chromatin domains that fine-tune transcription, replication, DNA damage repair, and faithful chromosome segregation. We developed computational and single-molecule nanoindentation tools to determine mechanical properties of histone variant nucleosomes. We found that the CENP-A nucleosome variant is more elastic than the canonical H3 nucleosome but becomes stiffer when bound to its partner CENP-C. In addition, CENP-C induces cross-array clustering, creating a chromatin state that is less accessible. These data suggest that innate material properties of nucleosomes can influence the ultimate chromatin state, and thereby influence biological outcomes.

The adaptive nature of chromatin allows a cell to replicate, divide, differentiate, regulate transcription, and repair damaged DNA. In part, the chromatin landscape is shaped by removing old and incorporating new nucleosomes with specific histone variants, and by incorporating covalent modifications (1–8). How different histone variants convey the unique mechanical properties of their nucleosomes to the chromatin fiber, and whether noncanonical nucleosomes modulate chromatin dynamics, is a subject of intense study. In contrast to the previous view that chromatin was a mostly static packaging polymer, several recent studies have unveiled a rich conformational landscape of nucleosomes (2). These works raise the intriguing possibility that mechanical properties embedded within evolutionarily distinct nucleosome types might lead to different structural outcomes for the chromatin fiber. Indeed, exciting advances in computational modeling have linked specific epigenetic chromatin modifications to chromosome architecture, genome folding, and genome dynamics (9–11). Paradoxically, the most evolutionarily divergent histone variant is CENP-A, which is functionally essential across most eukaryotes (12). Another major paradox is that despite being buried in pericentric heterochromatin (13–15), CENP-A chromatin is transcriptionally active in most species, suggesting that this chromatin is accessible even when bound to kinetochore proteins (16, 17). This puzzling dichotomy can be explained either by intrinsic mechanical properties or by epigenetic alterations driven by chromatin effectors.

To investigate this salient problem, we developed in silico and in vitro tools to dissect innate mechanical properties of CENP-A nucleosomes relative to their canonical counterparts, in the presence or absence of CENP-A binding partners, and extended these findings in vivo. We report that the smallest unit of the chromatin fiber can have profound effects on the 3D folding properties of chromatin, with implications for the accessibility of that chromatin to the transcriptional machinery.

## Results

### CENP-C^CM^ Increases the Young’s Modulus of CENP-A In Silico.

We first examined elasticity as a mechanical feature of nucleoprotein complexes, which has been previously unreported. Using all-atom molecular dynamics, we measured nucleosome stiffness and examined spontaneous structural distortions that occur in the presence of CENP-C. We ran 3 simulations for this study: 1) the CENP-A nucleosome core particle (NCP), 2) the CENP-A NCP with 1 bound rat CENP-C motif of CENP-C (CENP-C^CM^), and 3) the CENP-A NCP with 2 copies of CENP-C^CM^. As a control, we compared these systems to canonical nucleosomes, H3 (18).

**Fig. 1.**
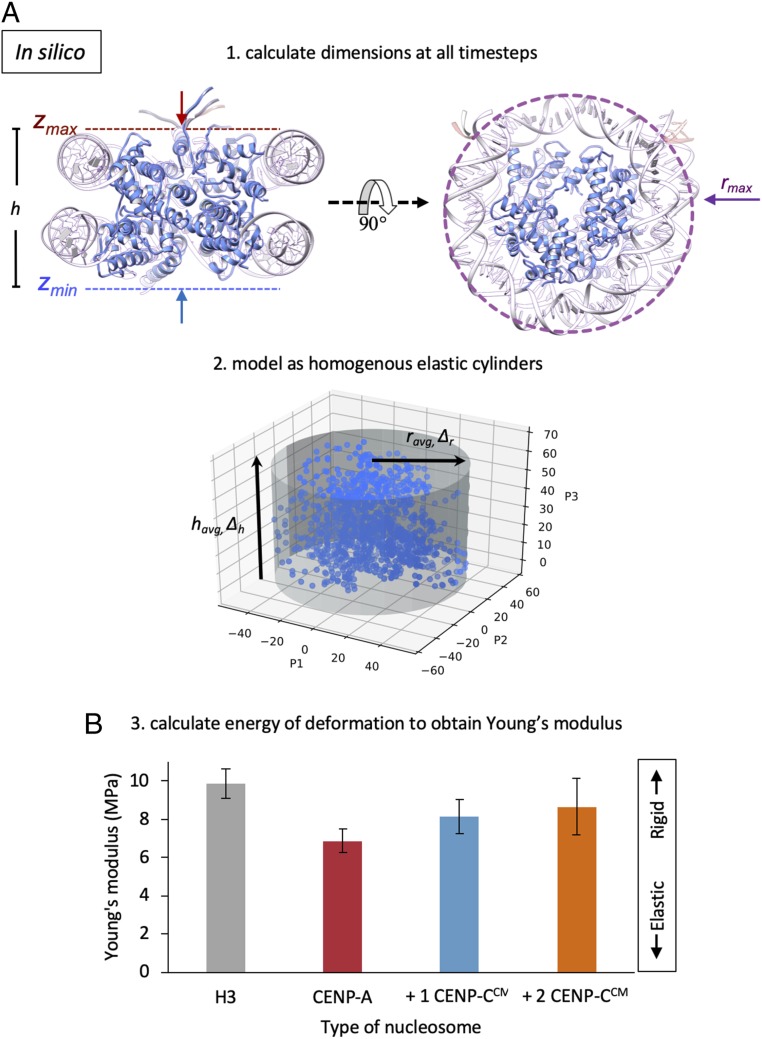
In silico analysis predicts that CENP-A nucleosomes are more elastic than H3 nucleosomes. (*A*) To obtain Young’s modulus values from simulation, we measured the in silico dimensions of nucleosomes by compression of an encapsulating cylinder programmed to stop at stiffer surfaces resistant to collapse. From the heights, *h* = *z*_max_ − *z*_min_, and the radii, *r*_max_, of the resulting minimal cylinders we then calculated the average and change in height (*h*_avg,_ Δ*h*), and radius (*r*_avg,_ Δ*r*) of each system. (*B*) We treated the nucleosomes as elastic homogenous cylinders, calculated the energy of deformation, and retrieved the Young’s modulus of a cylinder vibrating at equilibrium in a thermal bath.

Using these all-atom data, we next developed an analytical technique to quantify the elasticity of nucleosomes in silico (19). Briefly, this technique connects structural fluctuations observed in unbiased molecular dynamics simulations, with the nucleosome’s mechanical response, ultimately producing the absolute value of the Young’s modulus (*Methods*). To analyze all-atom simulation data in such a way, we modeled the nucleosomes as mechanically homogenous elastic cylinders vibrating in a thermal bath and calculated the dimensions and fluctuations of these “minimal” cylinders during each simulation trajectory (Fig. 1*A*). We further visualized the differences in fluctuations among different types of nucleosomes, finding a distinct height difference of the nucleosome core particle when bound to CENP-C^CM^ and an overall collapse in the variance of fluctuations (*SI Appendix*, Fig. S1 *A*–*D*). These analyses predict that the Young’s modulus of CENP-A is noticeably more elastic (6.2 ± 0.6 MPa) than that of H3 (9.8 ± 0.8 MPa). Interestingly, upon binding either 1 or 2 CENP-C^CM^ fragments (Fig. 1*B*), CENP-A nucleosomes stiffen (8.2 ± 0.9 MPa and 8.7 ± 1.5 MPa, respectively), nudging their elasticity profiles closer to that of H3. These findings speak to the flexibility of the multimeric nucleosome structure in comparison to its constituent parts. Indeed, in DNA stretching experiments, where DNA was pulled laterally, the Young’s modulus was found to be 3.3 GPa, while from a rod-bending model, 300 MPa was suggested (20). On the other hand, unrelated multimeric protein complexes, such as an antibody pentameter, were found to have Young moduli of 2.5 to 9 MPa (21), approximately in the same range as for the nucleosome core particles reported in this work.

### CENP-C Interactions Suppress Spontaneous Structural Distortions of CENP-A Nucleosomes.

The above discussed variation of elasticity of distinct nucleosomal complexes made us curious to examine conformational changes of CENP-A mononucleosomes that might be induced by CENP-C^CM^. To characterize the global motions of these complexes, we carried out principal component analysis (PCA), which is a method to identify larger amplitude and slower frequency motions ranked by variance (22, 23). These modes, which are akin to normal modes when analyzing molecular vibrations, are called the principal components (PCs). Subsequently, we obtained PCA free-energy (FE) plots through the histogramming of the first 2 PCs (*SI Appendix*, Fig. S1*E*). We found a somewhat rough FE landscape of the CENP-A nucleosome, similar to our previous studies on the origins of CENP-A’s intrinsic motions (18). However, upon binding of the CENP-C^CM^ fragment, which associates across the exterior of the histone core, the FE minima coalesce, showing a reduction in metastable configurations (*SI Appendix*, Fig. S1*E*). Furthermore, the PCA revealed a dampening of histone motions relative to each other upon binding of CENP-C^CM^ (*SI Appendix*, Fig. S1*E*), which is consistent with the above discussed elasticity observations.

We were next curious to assess how these changes would propagate through the DNA. Thus, we investigated DNA gyre sliding and gaping of nucleic acids through in silico labeling (*SI Appendix*, Fig. S2*A*). Indeed, a single CENP-C^CM^ fragment dampens CENP-A nucleosome gyre gaping and DNA slides asymmetrically away from the CENP-C^CM^ bound-face of CENP-A nucleosomes (*SI Appendix*, Fig. S2*A*). We performed additional structural analysis to demonstrate local structural flexibility. Altogether, detailed analyses of CENP-A mononucleosomes motions revealed a global dampening of innate motions upon CENP-C^CM^ binding (*SI Appendix*, Fig. S2*B*). On the residue scale, we found that CENP-C^CM^ suppresses residue fluctuations with symmetry breaking in the presence of 1 fragment (*SI Appendix*, Fig. S2*C*). These computational data are in agreement with experimental observations made by single-molecule fluorescence resonance energy transfer (sm-FRET) and hydrogen/deuterium-exchange mass-spectrometry (24–26) for the CENP-A nucleosome bound to the central domain region of human CENP-C (CENP-C^CD^). The CENP-C^CM^ and CENP-C^CD^ bind to CENP-A nucleosomes through the same mechanisms (27), likely because both domains contain the H2A/H2B acid patch binding motif [RR(S/T)nR] and the CENP-A C-terminal tail binding residues (WW/YW), which are separated by 7 residues. Importantly, these 2 motifs in CENP-C are conserved across plant, fungi, and animal kingdoms (*SI Appendix*, Fig. S3). These data predict that CENP-C dampens motions of CENP-A nucleosomes, and as a consequence, alters mechanical properties of the CENP-A nucleosome.

### CENP-A Nucleosomes Are More Elastic than H3 Nucleosomes In Vitro.

To experimentally test this prediction in vitro, we turned to nanomechanical force spectroscopy (28, 29). This single-molecule method is used to physically compress and release complexes to directly quantify their elasticity on a nanoscale (30–36). We were surprised to discover that the elasticity of nucleosomes has never been quantified. Therefore, we developed a protocol to perform in-buffer, single-molecule nanoindentation force spectroscopy of nucleosomes (*Methods*).

**Fig. 2.**
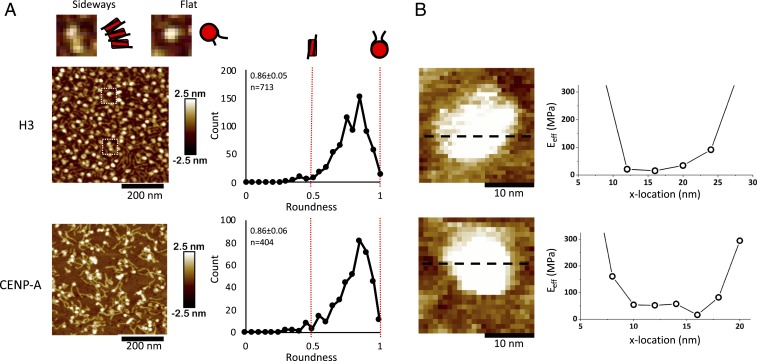
Mononucleosomes lie flat and are uniformly elastic. (*A*) Roundness was measured of either H3 or CENP-A mononucleosomes. A value of 1 would indicate that a particle lies flat on the mica surface and is perfectly round, whereas a value of 0.5 would indicate an oval shape, representing a nucleosome laying on its side. Almost all nucleosomal particles lie flat. Nucleosomes wrap 1.7 turns of DNA, which means that the exit and entry DNA strands are not on the same plane. As a result of this asymmetry, as well as the geometrical limitations of the AFM tip, the nucleosome becomes slightly wedge-shaped with a roundness value of 0.8. (Magnification, 2.5×.) (*B*) Young’s modulus was measured across individual H3 or CENP-A mononucleosomes to assess whether a nucleosome particle is uniformly elastic. No significant difference in Young’s moduli was observed across either nucleosome.

**Fig. 3.**
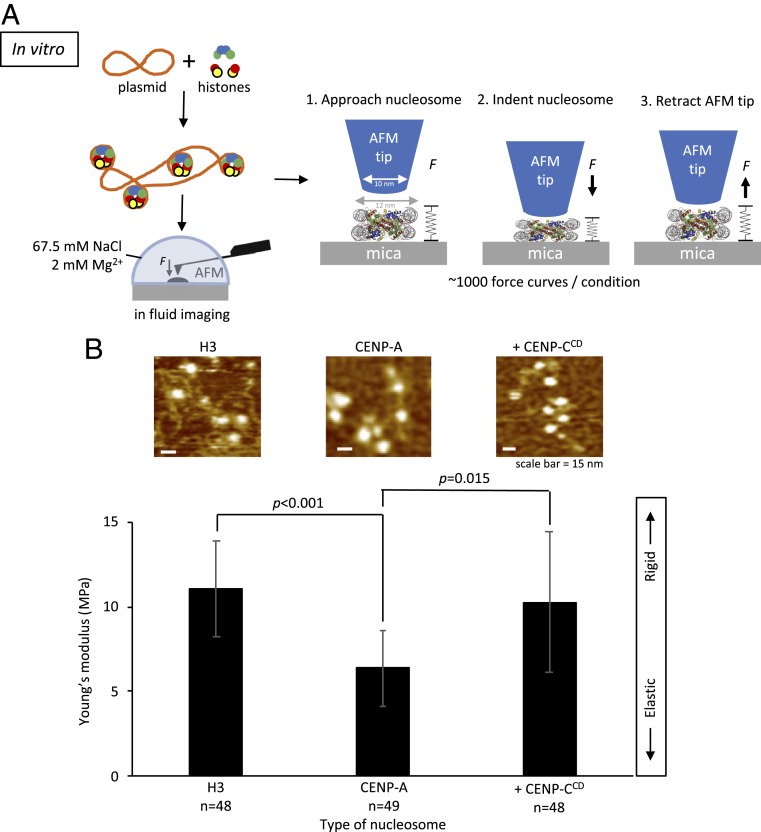
In vitro CENP-C^CD^ binding stiffens elastic CENP-A nucleosomes. (*A*) To determine the Young’s modulus of CENP-A and H3 nucleosome arrays, we in vitro-reconstituted H3 and CENP-A nucleosome arrays by salt dialysis. The AFM tip was aimed at the center of the nucleosome, which were indented by ∼1.5 nm under 150 to 200 pN of applied force. Nano-indentation force spectroscopy was performed under near physiological conditions. (*B*) Bar plot summarizing the Young’s modulus values showing that CENP-A nucleosomes are more elastic than H3 nucleosomes but become stiffer upon addition of CENP-C^CD^ (2-sided *t* test *P* < 0.0001). Approximately 1,000 force curves were measured per condition. Examples of individual force curves can be found in *SI Appendix*, Fig. S7.

Using traditional salt dialysis protocols (37, 38), we reconstituted H3 and CENP-A mononucleosomes on linear 187-bp DNA fragments (Fig. 2), or H3 and CENP-A nucleosome arrays on 3-kbp plasmids (Fig. 3*A*). To assess the quality of our reconstitutions, we determine nucleosomal dimensions by atomic force microscopy (AFM), as well as protection from nuclease (MNase) digestion. Consistent with previous work (39, 40), in fluid, in vitro reconstituted CENP-A nucleosomes possess dimensions similar to H3 nucleosomes (3.8 ± 0.3 and 3.7 ± 0.3 nm, respectively) (Table 1 and *SI Appendix*, Table S1). Similarly, nucleosome arrays yield classic nucleosomal ladders when challenged by MNase (*SI Appendix*, Fig. S4). Using mononucleosomes, we also established nucleosomal orientation, finding that nucleosomes almost always lay flat on mica (Fig. 2).

**Table 1.** Nanomechanical force spectroscopy indicates that CENP-C^CD^ stiffens and suppresses CENP-A nucleosomal elasticity

Nucleosome	*n*	FC	Young’s modulus (MPa)	Height (nm)	Diameter (nm)	Volume (nm^3^)
Mononucleosomes						
H3	5	24	35.4 ± 13.9	5.2 ± 0.53	11.3 ± 1.2	371 ± 107
CENP-A	4	34	18.5 ± 15.6	5.7 ± 0.53	11.7 ± 2.3	387 ± 86
Nucleosome arrays						
H3	48	997	11.3 ± 4.1	3.8 ± 0.3	14.0 ± 1.2	393 ± 68
CENP-A	46	977	5.8 ± 3.0	3.7 ± 0.3	13.7 ± 1.0	370 ± 61
+ 2× CENP-C^CD^	48	1000	9.4 ± 5.8	4.1 ± 0.5	13.5 ± 0.9	394 ± 61
+ 4× CENP-C^CD^	50	1014	15.2 ± 10.5	4.1 ± 0.6	14.0 ± 1.2	426 ± 61

Either H3 or CENP-A nucleosomes were in vitro-reconstituted on plasmid DNA and imaged in fluid in the presence or absence of 2-fold or 4-fold excess CENP-C^CD^. Values were rounded up to 1 decimal point. FC, number of force curves measured; *n*, number of nucleosomal particles measured. For each condition, at least 3 independent replicates were performed (Dataset S1).

Using these standardized nucleosomes, we then measured nucleosomal elasticity under near physiological conditions (67.5 mM NaCl, 2 mM Mg^2+^) (*Methods*). First, consistent with the computational model, CENP-A and H3 mononucleosomes display uniform elasticity across their surfaces, behaving as homogenous cylinders (Fig. 2). Second, individual CENP-A nucleosomes are twice as elastic compared to H3 nucleosomes (18.5 ± 15.6 MPa vs. 35.4 ± 13.9 MPa, respectively) (Table 1).

In vivo nucleosomes exist in arrays. Therefore, we extended these experiments to arrays of nucleosomes reconstituted on 601-containing plasmids under identical conditions (*Methods*). As noted above, MNase digestion and AFM measurements confirmed that nucleosome arrays were reconstituted efficiently (*SI Appendix*, Fig. S4). Remarkably, consistent with our computational results (Fig. 1*B*) and with the result for mononucleosomes (Table 1), the effective Young’s moduli of H3 and CENP-A nucleosomes are distinct. The Young’s modulus of H3 nucleosomes is 11.3 ± 4.1 MPa, whereas CENP-A nucleosomes are nearly twice as elastic, at 5.8 ± 3.0 MPa (Fig. 3*B* and Table 1).

### CENP-C^CD^ Stiffens CENP-A Nucleosomes In Vitro.

Our in silico experiments predicted that CENP-C^CM^ suppresses CENP-A nucleosomal motions and consequently innate elasticity (Fig. 1). We tested this prediction in vitro. We first examined the behavior of CENP-A nucleosomes in the presence of human or rat CENP-C^CM^. We observed a qualitative increase in cross-array clustering of CENP-A chromatin arrays (*SI Appendix*, Fig. S5). This rapid clustering by the CENP-C^CM^ fragment made it challenging to measure the rigidity of individual nucleosomes reliably. To resolve this challenge, we continued our investigation with CENP-C^CD^ which, as noted above, has the conserved binding motif of CENP-C^CM^ (*SI Appendix*, Fig. S3). The addition of human CENP-C^CD^ resulted in a 0.4-nm height increase of CENP-A nucleosomes (3.7 ± 0.3 nm vs. 4.1 ± 0.5 nm) (Table 1 and *SI Appendix*, Figs. S6 and S7 and Table S1), lending confidence that CENP-C^CD^ is bound to CENP-A nucleosomes.

Next, we measured the Young’s moduli of CENP-C bound vs. free CENP-A nucleosomes (*Methods*). With the addition CENP-C^CD^ at 2-fold excess, we observed that half the CENP-A nucleosomes remained highly elastic (∼5 MPa), but the other half lost elasticity by a factor of 3 (∼14.5 MPa) (Fig. 3*B* and Table 1) (*t* test *P* = 0.015). One obvious interpretation of this distribution is that it arises from 2 distinct CENP-A subspecies: Unbound and flexible vs. bound and rigidified by CENP-C. To test this idea, we doubled the amount of CENP-C^CD^ to 4-fold excess. Under these conditions, virtually all CENP-A nucleosomes become stiffer (15.2 ± 10.6 MPa) (Table 1 and *SI Appendix*, Figs. S7 and S8).

These data show that in silico and in vitro CENP-A nucleosomes possess innate elasticity and that CENP-C effectively suppresses the freedom of motions of CENP-A nucleosomes. From a thermodynamic perspective, elastic particles possess higher configurational entropy (41–43). In other words, elastic particles tend to be less ordered. Thus, we were curious to test whether nucleosomes with a broadened range of configurational states might collectively form less ordered chromatin and energetically disfavor compaction.

### CENP-C Induces Cross-Array Clustering In Vitro, Ex Vivo, and In Vivo.

**Fig. 4.**
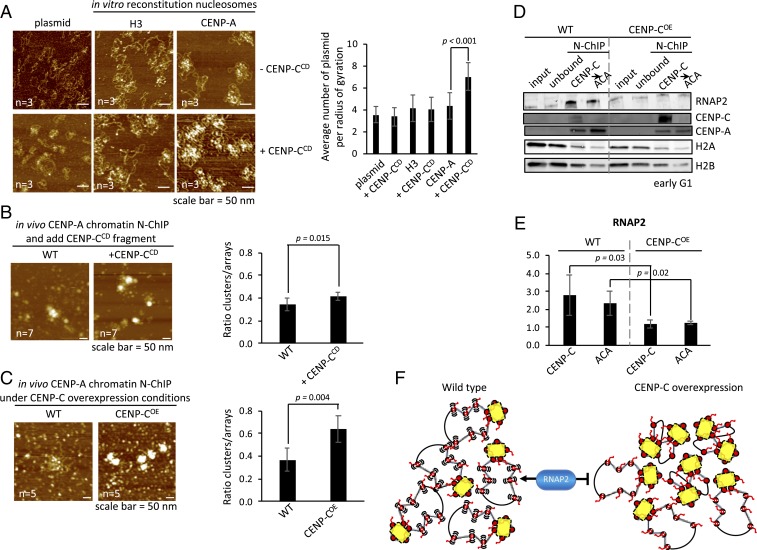
CENP-C overexpression compacts CENP-A chromatin, making it inaccessible to RNAP2. (*A*) Quantitative assessment of in vitro reconstituted chromatin showed that only CENP-A chromatin clustered in the presence of CENP-C^CD^ fragment, whereas H3 chromatin or naked plasmids did not in the presence of CENP-C^CD^. Plasmid clustering was measured by counting the number of plasmids in a radius of gyration (*r* = 0.25 µm). (*B*) To determine if the CENP-C^CD^ fragment used in the in vitro experiments could induce CENP-A chromatin compaction, we added CENP-C^CD^ for 30 min to isolated free CENP-A chromatin from HeLa cells. Nucleosome arrays can be identified as either bead-on-a-string or large compacted clusters where DNA strands can be seen entering/exiting. Compacted chromatin was scored over the total number of nucleosome arrays. (*C*) Similar analysis was performed on unbound CENP-A chromatin extracted from cells that either did (CENP-C^OE^) or did not (WT) overexpress CENP-C. (*D*) Centromeres are expressed during early G1. Therefore, we synchronized HeLa cells to early G1 and extracted kinetochore-bound (first CENP-C N-ChIP) and unbound CENP-A chromatin (second ACA N-ChIP of unbound fraction; see *Methods* for details). By Western blot we probed for RNAP2, CENP-C, CENP-A, H2A, and H2B. (*E*) Quantification of RNAP2 levels were determined by LiCor’s software. The bar graphs represent 3 independent experiments. (*F*) Working model of CENP-C (yellow) overexpression inducing CENP-A chromatin (red) cross-array clustering thereby reducing access to RNAP2 (blue).

We first sought to tease out this idea by incubating in vitro reconstituted CENP-A chromatin arrays with or without CENP-C^CD^ and observed these arrays by in-air AFM. Upon addition of CENP-C^CD^, CENP-A arrays demonstrated a 1.6-fold increase in cross-array clustering (Fig. 4*A*). This clustering was not observed for controls, namely CENP-C^CD^ incubated with either H3 chromatin or naked DNA (Fig. 4*A*).

We next tested whether ex vivo, kinetochore-depleted CENP-A chromatin purified from human cells (*Methods*) (44) would cluster solely upon the addition of recombinant CENP-C^CD^ (Fig. 4*B*). Relative to free CENP-A chromatin, we observed a modest 1.2-fold increase in chromatin clusters upon CENP-C^CD^ incubation (34 ± 6% vs. 42 ± 4%, 2-sided *t* test *P* = 0.015) (Fig. 4*B* and *SI Appendix*, Table S2).

A logical hypothesis arising from these in vitro and ex vivo results is that excess CENP-C induces a more compact CENP-A chromatin state in vivo. To test this idea, we overexpressed full-length CENP-C (CENP-C^OE^) in human cells for 3 d, after which we purified kinetochore-depleted CENP-A chromatin by serial native chromatin-immunoprecipitation (N-ChIP) (*Methods*) (44). We quantified native CENP-A chromatin clusters using the same method as above. Upon CENP-C^OE^, we observed a nearly 2-fold increase in chromatin clusters relative to the wild-type control (37 ± 10% vs. 64 ± 11%, 2-sided *t* test 0.004) (Fig. 4*C* and *SI Appendix*, Table S2). Thus, in vitro, ex vivo, and in vivo, CENP-C increases the population of CENP-A chromatin clusters.

### CENP-C Overexpression Limits Centromeric Chromatin Accessibility In Vivo.

It has been demonstrated that chromatin accessibility is prognostic of transcriptional competency across the genome (45, 46). This correlation was first reported decades ago in 2 seminal works demonstrating nuclease hypersensitivity of actively transcribing loci (47, 48). We hypothesized that an innately open CENP-A chromatin state would be accessible, whereas excess CENP-C should reduce the accessibility of CENP-A chromatin in vivo. One read-out of altered compaction status would be reduced accessibility of CENP-A chromatin to transcriptional machinery.

To test this idea, we performed CENP-C^OE^ for 3 d and synchronized the cells to early G1, when centromeres are transcribed in human cells (16, 17, 49). From these cells, we purified CENP-C bound centromeric chromatin as well as any residual CENP-A chromatin by serial N-ChIP (*Methods*) (44). We assessed the occupancy of active RNA polymerase 2 (RNAP2) on these purified native chromatin arrays from wild-type or CENP-C^OE^ cells. By Western blot analysis, when CENP-C is overexpressed, we observed a significant reduction in RNAP2 levels on centromeric chromatin (3- and 2-fold reduction, respectively; 2-sided *t* test *P* < 0.05) (Fig. 4 *D* and *E* and *SI Appendix*, Table S3). Thus, CENP-C overexpression leads to both, CENP-A chromatin clustering and reduced accessibility of transcriptional machinery.

These data show that CENP-C overexpression suppresses accessibility of centromeric chromatin (Fig. 4*F*), correlating with the attenuation of transcriptional machinery.

## Discussion

Not all nucleosomes are identical, as many contain histone variants, giving them distinct structures and functions (1, 2, 6). In this report, we systematically teased apart how a single histone variant encodes mechanical properties to its nucleosome, which were dramatically modified by a small fragment of its cognate protein partner. Using in silico computational modeling and in vitro single-molecule nanoindentation force spectroscopy, we directly measured effective elasticity of nucleosomes and found that CENP-A is more elastic than canonical nucleosomes (Figs. 1–3 and Table 1). Indeed, we found remarkable agreement between the computation model to derive the Young’s modulus, and the experimental data measuring the elasticity. Second, our findings of noticeably elastic CENP-A nucleosomes (19) have important implications. On the 1 hand, softer CENP-A nucleosomes are expected to undergo more vigorous structural fluctuations, in turn, potentially exposing cryptic binding surfaces that may facilitate various association outcomes. On the other hand, softer CENP-A nucleosomes may contain excess entropy compared to canonical nucleosomes, which, in turn, would suggest an additional entropy loss upon formation of compacted CENP-A chromatin. However, dynamics of different histone variants are difficult to predict a priori: For example, H3 nucleosomes within HP1 chromatin show, surprisingly, increased dynamic behavior (50, 51). Therefore, although we anticipate additional entropic resistance to compaction for chromatin enriched with CENP-A nucleosomes, it will be exciting to apply tools developed in this work in future studies for other important types of nucleosomal complexes.

CENP-C is the essential CENP-A binding protein, which facilitates the assembly of the kinetochore (52–54), and has been shown to alter local CENP-A nucleosomes dynamics (24–26). Previous FRET and hydrogen/deuterium exchange mass spectrometry experiments focused on how CENP-C^CD^ binding alters internal CENP-A mononucleosome dynamics. These data show that human CENP-C^CD^ restricts DNA gyre gaping, sliding, and protects the internal H4/H2A interface (24–26). In our prior computational modeling, we showed that CENP-A nucleosomes sample broadened conformational states (18). From this, we predicted that CENP-C limits configurations of CENP-A nucleosomes. Indeed, when we modeled CENP-A nucleosomes alone vs. those bound to CENP-C^CM^, we observed marked diminution of nucleosome motions, and increased Young’s moduli, representing lost conformational flexibility (Fig. 1*B* and *SI Appendix*, Fig. S2). Direct elasticity measurements by nanoindentation force spectroscopy revealed that CENP-C^CD^ stiffens the CENP-A nucleosomes in a dose-dependent manner (*SI Appendix*, Fig. S8). A physical analogy is that CENP-C behaves as a nanoscale staple on the surface of the CENP-A nucleosome, inhibiting intra- and intermolecular motions and propagates these to the chromatin fiber. Furthermore, the homodimerization domain of CENP-C likely exaggerates cross-array clustering (55). Thus, a speculative prediction from this model is that the centromeric fiber harbors a free CENP-A domain to allow cell-cycle regulated transcription of centromeres, required for the loading of new centromeric proteins (16, 17, 49).

An alternative plausible hypothesis is that CENP-C binding might change CENP-A internucleosomal stacking potential in an array. In addition to CENP-A’s C-terminal domain, CENP-C is known to bind the H2A/H2B acidic patch (27, 56, 57). Indeed, the H2A/H2B acidic patch has been long recognized as an important nucleosomal site for docking chromatin effector proteins (2). To date, nucleosome stacking studies have exclusively focused on H3 nucleosomes, which revealed that stacking is mediated through the N-terminal tail of H4 interacting with the H2A/H2B acidic patch of a neighboring nucleosome (58). Two recent studies showed that CENP-C^CD^ alters the direction of the N-terminal tail of H4 within CENP-A mononucleosomes (56), which can be further fixed by CENP-N binding (57). Thus, these results would suggest that CENP-C impedes CENP-A nucleosome stacking. That said, given the 3D boustrephedon-like folding structures observed in superresolution microscopy studies of vertebrate centromeres (85), cross-array interdigitated clustering remains an intriguing possibility that bears exploration in future work.

We note that CENP-C expression is tightly regulated, despite overexpression of many centromere proteins in human cancers, including CENP-A (39, 60–62). Taken in the context of our findings in this report, maintaining the correct ratio between CENP-A and CENP-C in vivo might be critical for preserving the mechanical features of centromeric chromatin. In human cancer cells, where CENP-A is overexpressed and ectopically localized to subtelomeric breakpoints (39, 60), 1 unexpected mechanical outcome might be the induction of large swathes of elastic CENP-A chromatin at inappropriate regions of the genome (63). This will be an exciting avenue to pursue in future studies.

Centromeric DNA and centromeric protein genes are rapidly evolving (15, 64–69). Not all species share all kinetochore components: Centromeric genes are lost, duplicated, and sometimes invented (70–72). Despite these evolutionary changes, the distinctive chromatin structure of centromeres must be maintained. Investigating whether CENP-A elasticity is a feature arising from its surprising rapid evolution, or whether it is conserved and coevolved with kinetochore proteins, will shed light on centromeric evolution. Thus, even at the level of its nanoscale components, the centromere serves as an excellent model to study the evolution of epigenetic systems.

## Methods

### All-Atom Computational Modeling.

We built 3 nucleosomal systems for simulation: the CENP-A nucleosome, as described previously (73), and the CENP-A nucleosome with 1 and 2 rat CENP-C motif (CENP-C^CM^) fragment bound from PDB ID code 4X23 (27). The CENP-C^CM^ fragments were docked onto the CENP-A interface using the CE algorithm (74) of PyMOL (The PyMol Molecular Graphics System). All-atoms molecular dynamics simulations were performed with software suite GROMACS 5.0.4 (75). The force field employed to model nucleosomes was amber99SB*-ILDN (76, 77) for proteins, amber99SB parmbsc0 (76) for DNA, ions08 (79) for ions, and the TIP3P water model. Unresolved 3AN2 residues Thr-79 through Asp-83 of CENP-A′, Chain E, were built with the program MODELER (80). During energy minimization of this constructed region, 1 residue in the N terminus and C terminus directions were unconstrained. Additionally, selenomethionine residues were altered to methionine through a single-atom mutation from Se to S. As a control, the 146-bp α-satellite DNA (81) was aligned onto 3AN2 using the CE algorithm (74) of PyMOL (82). Systems 2 and 3 were built by docking the CENP-C^CM^ fragment from the recently solved structure of an H3 chimera nucleosome bound to CENP-C^CM^ onto the final 1-μs snapshot of simulation 1, which was then subsequently run for an additional microsecond.

From these initial structures, the GROMACS tool pdb2gmx was used to assign charges to residues at biological pH. Then, a rectangular cuboid box was created such that boundaries were a minimum distance of 1.5 nm from the unsolvated system. Next, Na^+^ and Cl^−^ ions were introduced to neutralize the system charge and model an ionic physiological concentration to 150 mM NaCl. For both preproduction and production runs, periodic boundary conditions were employed. Electrostatics were handled with the Particle Mesh Ewald method and Verlet cutoff scheme. For the nonbonded interaction shift functions, Coulombic and Van der Waals potentials had a cutoff distance at 1.0 nm. Hydrogen bonds were constrained with the LINCS algorithm.

Each system was energy-minimized using steepest descent to a maximum energy of 100 kJ/mol. The systems were then equilibrated in multiple steps. First, the systems were heated to 300 K for 2,000 ps. During this step, DNA was restrained with *K* = 1,000 kJ mol^−1^ nm^−2^ in the Canonical ensemble (NVT). For the next thermal equilibration at 300 K for 2,000 ps, both DNA and protein had weak harmonic position restraints *K* = 2.5e-5 kJ mol^−1^ nm^−2^ to prohibit large nucleosome rotations. Finally, pressure was equilibrated for 1,500 ps in the isothermal-isobaric, NPT, ensemble at 300 K and 1.0 bar.

Production simulations were performed for 1 μs at 300 K. Temperatures were V-rescaled with the modified Berendsen thermostat (83) with a time constant of 1.0 ps. System pressures were regulated with the Parrinello–Rahman barostat (84) at 1.0 bar and a time constant of 2.0 ps. The simulations’ time-step size was 2 fs. Coordinates, velocities, and energies were saved every 2 ps. Nonbonded neighbors lists were updated every 20 fs. For subsequent analysis, trajectories were truncated to remove the first 600 ns to account for additional system equilibration during production runs. We performed our structural analysis calculations, PCA, contact analysis, and root mean square fluctuation (RMSF), as described previously (73).

For quality control and checks for equilibration, we checked the energy minimization, equilibration, and running RMSD for the simulations (*SI Appendix*, Fig. S9). Both CENP-A and CENP-A with 1 and 2 CENP-C^CM^–bound (27) ran for an additional microsecond and the first 600 ns of simulation time were truncated from the dataset for further analysis and to account for equilibration. For a control to compare to this dataset, we also analyzed the H3 nucleosome from our previous work (18). In addition to our prior description, after energy minimization we checked our structures for potential clashes based on van der Waals radii through the accepted range of 0.4 to 1.0 Å and verified that there were no clashes in the nucleosome structures.

### Computational Calculations of Gaping and Sliding.

Furthermore, we calculated the relative positions of 3 phosphate backbone atoms at positions −33, −43, and +38 numbered from the 5′ (−) to 3′ (+) direction relative to the pseudodyad as previously marked in FRET experiments to measure gaping and sliding (26). The distances between these points and the skew of the triangle formed were measured and then plotted with the initial position of residue −33 set to (0,0) on an *x-y* plane. The distribution of Δ*y* and Δ*x* of +38 relative to −33 and −34 was used to measure DNA gaping and sliding, respectively. We visualized these distributions with standard box plots showing the mean, the interquartile range, and whiskers extending to the extrema. The distribution of polygons contains the minima and maxima of all 3 vertices were plotted visually with triangles to present changes in skew and the range of sizes. Comparative shifts in DNA motions toward gaping and sliding were used to show a trend toward those motions, but with lesser magnitude of motion compared to experiments, since the simulation timescale is orders-of-magnitude smaller than the corresponding experimental time scales.

### In Silico Calculation of Young’s Modulus.

The goal of this analysis is to model each nucleosome as a homogenous elastic “minimal” cylinder for each time step of the simulation, retrieve the cylinder height and radius distributions, and from these data calculate the in silico Young’s modulus of the nucleosomes. Our method to calculate the dimensions of the minimal cylinders follows the workflow:

First, orient the nucleosomes so that they lie “flat” on the *x-y* plane. To achieve this, we calculated the principal axes of the moment of inertia, where the first principal axis defines the broadest plane of the nucleosome. The axes of symmetry of the nucleosomes align with the 3 principal axes, p_1_, p_2_, p_3_, with the center-of-mass at the origin.

Second, calculate the surfaces of the cylinder so that they coincide with stiffer regions of the nucleosomes. We addressed this issue by calculating the RMSF of each residue along the simulation since the structural disorder of a region positively correlates with local structural fluctuations. Since RMSF is a time-averaged parameter, multiple time steps are required to calculate fluctuations of residues. As a result, we divided the simulation into windows (800 windows per simulation) and calculated the RMSF for each residue in each window.

Third, retrieve the average heights, radii, and the variances of these distributions. To do so, we sorted the C-α coordinates by their *z* axis coordinates and selected the *z* coordinate of the residue where 10 stiffer residues below an RMSF threshold were excluded outside of the cylinder volume. From the height, *h*, and radius, *r*, data we calculated the average *h* and *r*, the variance or spread of the distributions, and the SDs Δ*r* and Δ*h*.

Fourth, the outputs from step 3 then served as the variable inputs to calculate the Young’s modulus of each system. The work done in the deformation of an elastic material is stored in the form of strain energy, which we calculate for the deformation of the cylinder in the absence of the shear stresses. In our simulations, the amplitude of vibrations depends on the amount of energy given to the system from the temperature, or the thermal bath of the solvent. From equipartition theorem, 1/2 *k*_b_*T* (where *k*_b_ is the Boltzmann constant and *T* is temperature, 300 K) is the amount of energy attributed to the observed cylinder deformation. From the data on the average cylinder conformation, the magnitude of elastic deformation, and the energy input from the thermal bath we calculate the Young’s modulus. We calculated the SD of Young’s modulus values from 3 independent subsections of the analyzed trajectories.

### Single-Molecule Nanoindentation Force Spectroscopy of Mononucleosomes.

H3 (H3 mononucleosome on 187 bp of 601 sequence cat#16-2004, EpiCypher) and CENP-A mononucleosome (CENP-A/H4 cat#16-010, H2A/H2B cat#15-0311, 187 bp of 601-sequence cat#18-2003, EpiCypher) samples were diluted 1:5 in 2 mM NaCl with 4 mM MgCl (pH7.5) and deposited onto freshly cleaved mica that had previously been treated with aminopropyl-silantrane (APS), as described previously (40, 85, 86). Samples were incubated on mica for ∼3 min, excess buffer was rinsed with 400 μL ultrapure, deionized water, and gently dried under an argon stream. Imaging was performed with a commercial AFM (MultiMode-8 AFM, Bruker) using silicon-nitride, oxide-sharpened probes (MSNL-E with nominal stiffness of 0.1 nN/nm, Bruker). Deposited sample was rehydrated with 10 mM Hepes (pH 7.5), 4 mM MgCl_2_. Imaging was performed in AFM mode termed “Peak-Force, Quantitative NanoMechanics” or PF-QNM. Images were preprocessed using the instrument image analysis software (Nanoscope v8.15) and gray-scale images were exported to ImageJ analysis software (v1.52i). First, nucleosomes were identified as described previously (40, 86), and subsequently roundness was determined. The Young’s modulus was determined by the instrument image analysis software (Nanoscope v8.15).

### Optimization of Single-Molecule Nanoindentation Force Spectroscopy.

Nucleosomes that lie flat have a round appearance, whereas nucleosomes lying on their side would have an oval appearance. We measured the roundness of both H3 and CENP-A mononucleosomes and found that almost all nucleosomes had a round appearance (Fig. 2*A*).

The use of AFM nanoindentation of nucleosomes raise 2 more concerns. One is that the size of the probe is of the same order-of-magnitude as the nucleosome. Therefore, widely used, Hertz-type models used to extract elasticity from indentation data would only provide an effective elasticity that depends on the indentation geometrical parameters, such as probe size and precise point of indentation on the nucleosome. This effective elasticity would, however, be comparable between the 2 types of nucleosomes and their relative values would be comparable to those obtained in silico. The probe sizes used did not vary significantly but we needed to address the possibility that the extracted elasticity depends strongly on the exact point of indentation. If the nucleosome is not uniformly elastic, the precise position of the AFM probe tip could be a critical factor. If the nucleosomes are uniformly elastic, slight differences in where on the nucleosome the elasticity is measured would not be a major concern. We therefore measured the Young’s modulus across mononucleosomes (Fig. 2*B*). As we are working close to the limit of the instruments noise floor, we considered a Young’s modulus variation below an order-of-magnitude as acceptable. Indeed, measurements of the surrounding mica did show variability greater than an order-of-magnitude. We found that, in general, effective elasticity did not vary significantly across nucleosomes (Fig. 2*B*).

### Single-Molecule Nanoindentation Force Spectroscopy of Nucleosome Arrays.

In vitro reconstitution of CENP-A nucleosome arrays (CENP-A/H4 cat#16-010 and H2A/H2B cat#15-0311, EpiCypher) and H3 (H3/H4 cat#16-0008 and H2A/H2B cat#15-0311, EpiCypher) on a 3-kb plasmid containing a single 601 sequence (pGEM3Z-601 from Addgene #26656) were performed as previously described (40, 86). A human CENP-C_482–527_ fragment (CENP-C^CD^) (27) and rat CENP-C_710–740_ (CENP-C^CM^) (ABI Scientific) was added in 2.2-fold or 4-fold molar excess to CENP-A nucleosomes. Imaging was performed by using standard AFM equipment (Oxford Instruments, Asylum Research’s Cypher S AFM). To be able to measure the Young’s modulus, the reconstituted chromatin was kept in solution containing 67.5 mM NaCl and 2 mM Mg^2+^ and Olympus microcantilevers (cat# BL-AC40TS-C2) were used. Before each experiment, the spring constant of each cantilever was calibrated using both GetReal Automated Probe Calibration of Cypher S and the thermal noise method (87). Obtained values were in the order of 0.1 N/m. As a reference to obtain the indentation values, the photodiode sensitivity was calibrated by obtaining a force curve of a freshly cleaved mica surface. All experiments were conducted at room temperature. Force-curves for ∼50 nucleosomes for all 3 conditions were measured using both “Pick a Point” and force-mapping mode. The maximum indentation depth was limited to ∼1.5 nm and the maximum applied force was 150 to 200 pN. For our analyses, we used a Hertz model with spherical indenter geometry for Young’s modulus measurements, δ = [3(1 − *ν*^2^)/(4*ER*^1/2^)]^2/3^*F*^2/3^ (for a spherical indenter), where *ν* is the Poisson ratio of the sample, which is assumed to be one-third as in studies reported previously (32, 35); *δ*, *F*, *E*, and *R* are the indentation, force, Young’s modulus of the sample and radius of the tip, respectively. The radius of the tip was confirmed by SEM and found to be about 10 nm in width. Graphs were prepared using ggplot2 package for R.

### AFM and Cluster Analysis.

Imaging of CENP-C and CENP-A N-ChIP and bulk chromatin was performed as described previously (40, 86) with the following modifications. Imaging was acquired by using commercial AFM equipment (Oxford Instruments, Asylum Research’s Cypher S AFM) with silicon cantilevers (OTESPA or OTESPA-R3 from Olympus with nominal resonances of ∼300 kHz, stiffness of ∼42 N/m) in noncontact tapping mode or commercial AFM (MultiMode-8 AFM, Bruker) using silicon cantilevers (OTESPA or OTESPA-R3 from Olympus). In vitro samples were exposed to either 1) rat or 2) human CENP-C^CM^ or 3) human CENP-C^CD^ fragments, whereas in vivo samples were only exposed to human CENP-C^CD^. In vitro samples were 1) naked plasmid DNA, 2) reconstituted H3, or 3) reconstituted CENP-A chromatin. In vivo samples were kinetochore-depleted chromatin obtained from HeLa cells, as described previously (44). All samples were incubated for with the CENP-C fragment for 30 min at room temperature on an end-over-end rotator, before being deposited on freshly cleaved mica. HeLa cells which transiently transfected with CENP-C were used to isolate kinetochore-depleted chromatin. APS-mica was prepared as previously described (40, 86). The samples were incubated for 10 min, gently rinsed with 2× 200 μL ultrapure water, and dried with inert argon gas before imaging. Plasmid clustering was quantified by counting the total number of plasmids in a 0.25-µm radius of gyration around grouped plasmids. To quantify chromatin compaction, we manually counted chromatin clusters based on their size being at least twice as wide as an individual nucleosome, but with an identifiable entry and exit DNA strand. The cluster was counted over the total number of nucleosome arrays (clustered and not clustered).

### N-ChIP and Western Blotting.

Human cell line HeLa were grown in DMEM (Invitrogen/ThermoFisher Cat #11965) supplemented with 10% FBS and 1× penicillin and streptomycin mixture. N-ChIP experiments were performed without fixation. After cells were grown to ∼80% confluency, they were harvested as described previously (35, 44, 73), but with a few modifications. These were that all centrifugation was done at 800 or 1,000 rpm at 4 °C. Chromatin was digested for 6 min with 0.25 U/mL MNase (Sigma-Aldrich cat #N3755-500UN) and supplemented with 1.5 mM CaCl_2_. The first N-ChIP was with 5 μL guinea pig CENP-C antibody, subsequently the unbound fraction was subjected to N-ChIP with 5 μL anticentromere antibody (ACA) serum (*SI Appendix*, *Methods*). For CENP-C overexpression we transfected HeLa cells with pEGFP-CENP-C using the Amaxa Cell Line Nucleofector Kit R (Lonza cat#VVCA-1001) per the manufacturer’s instructions. HeLa cells were synchronized to early G1 by double thymidine block (0.5 mM, Sigma-Aldrich cat#T9250). After the first block of 22 h, cells were released for 12 h, followed by a second thymidine block of 12 h. Cells were released for ∼11 h, which corresponds to early G1, based on our previous reports (35, 73).

### Quantification and Statistical Analyses.

Significant differences for nucleosome height measurement from AFM analyses and significant differences for immunostaining quantification and chromatin compaction quantification were performed using the 2-sided *t* test, as described in the figure legends and text. Significant differences for the Young’s modulus of in vitro reconstituted H3, CENP-A, and CENP-A + CENP-C^CD^ were determined using a 1-way ANOVA test using GraphPad Prism software. Significance was determined at *P* < 0.05.

### Data Availability.

Code used to determine the Young’s modulus from all-atom computational modeling can be found here: https://github.com/pitmanme/pitmanme.github.io.

## Supplementary Material

Supplementary File

Supplementary File
