# Supplementary material for: Intrinsic elasticity of nucleosomes is encoded by histone variants and calibrated by their binding partners
Source: Proc Natl Acad Sci U S A. 2019 Nov 11;116(48):24066–74. doi: 10.1073/pnas.1911880116 (PMC6883791; doi:10.1073/pnas.1911880116)
Supplement: Supplementary File [file pnas.1911880116.sapp.pdf]

SI Appendix, Methods

Key Resources Table

Antibody	Source	Identifier	Application	Quantity
ACA serum	BBI Solutions	SG140-2	N-ChIP	5 μL
α-CENP-A (mouse)	Abcam	ab13939	IF	1:1000
α-CENP-A (rabbit)	Milipore	04-205	WB	1:3000
α-CENP-C (guinea pig)	MBL International	PD030	N-ChIP	5 μL
α-CENP-C (rabbit)	Santa Cruz	sc-22789	IF, WB	1:1000; 1:500
α-RNA polymerase II	Abcam	ab5095	WB	1:500
α-H2A	Abcam	ab18255	WB	1:1000
α-H2B	Abcam	ab1790	WB	1:1000

IF = immunofluorescence; N-ChIP: native chromatin immunoprecipitation, WB = western blot

Software and Algorithms	
Gwyddion	http://gwyddion.net/
NanoScope	http://www.nanophys.kth.se/nanophys/facilities/nfl/afm/icon/bruker-help/Content/SoftwareGuide/NanoScope815CoverPage.htm
Asylum Research	Version 15
Igor Pro	https://www.wavemetrics.com/taxonomy/term/87
R	https://www.r-project.org/
ggplot2	https://ggplot2.tidyverse.org/
LiCor Image Studio	https://www.licor.com/bio/products/software/image_studio_lite/
NIH ImageJ	https://imagej.nih.gov/ij/
GraphPad Prism 8	https://www.graphpad.com/
Bio-Formats	https://www.openmicroscopy.org/bio-formats/
PyMOL	https://pymol.org/2/
MEME	http://meme-suite.org/tools/meme

Contact for Reagent and Resource Sharing

Requests for further information or reagents should be directed to the Lead Contacts:

Yamini Dalal (dalaly@mail.nih.gov) and Garyk Papoian (gpapoian@umd.edu).

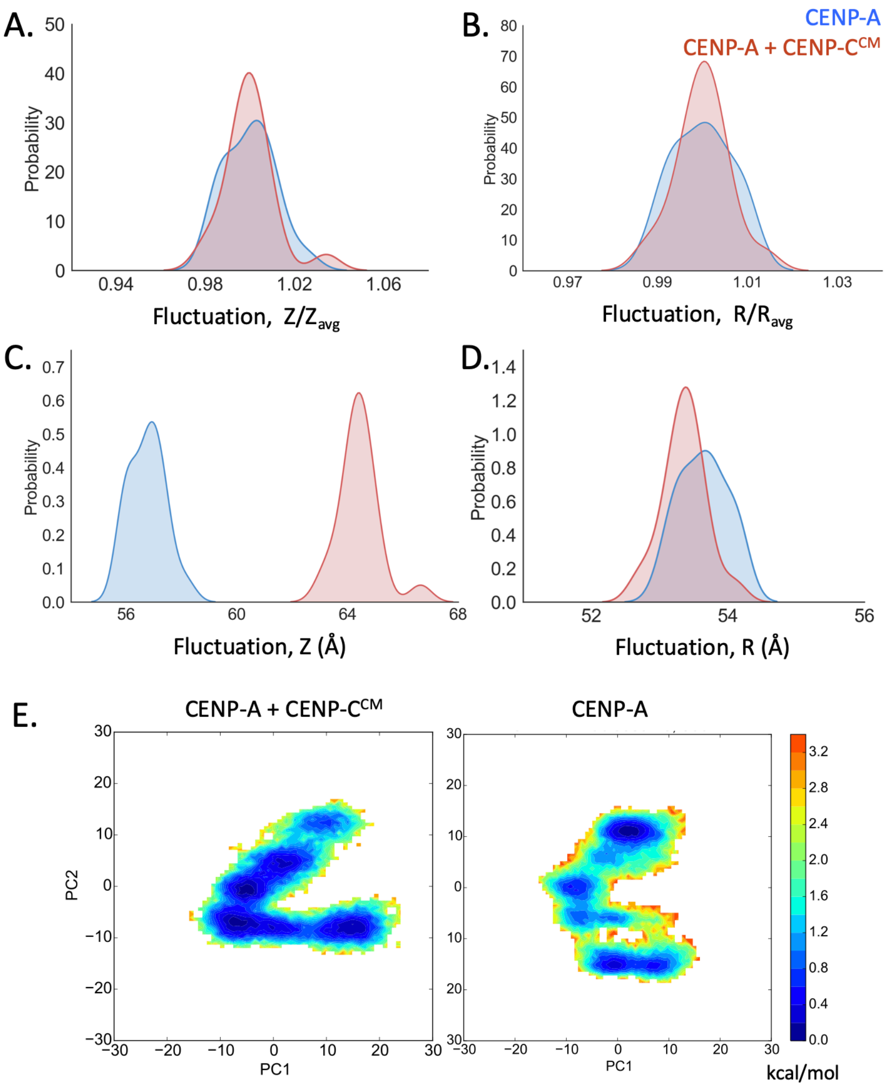

Figure S1 – CENP-C^{CM} limits CENP-A nucleosomal fluctuations
Raw data of the calculation of Young's modulus are shown for CENP-A and CENP-A + CENP-C^{CM} for comparison. CENP-A is shown in blue and CENP-A + CENP-C^{CM} in red. (A) The ratio of the measured height at each simulation segment to the average nucleosome height is shown. (B) Raw data for radial distributions is shown. (C) Height measurements are shown and reveal a distinct difference in the heights of the nucleosome core particles. The CENP-C^{CM} fragment is excluded from height measurements. (D) Radial measurements show increased fluctuation for CENP-A but with similar dimensions. The fluctuations are calculated from the variance of plots (C) and (D) and the Young's modulus derived from the strain energy density is sensitive to volume, such that the same magnitude fluctuation on a larger structure obtained increased values of Young's modulus. (E) The projection of the first two principal components (PC1 and PC2) are shown to obtain coarse grained free energy landscapes. Free energy minima are shown in blue and less frequently sampled conformations correspond to red. CENP-A shows a glassier free energy landscape with higher energetic barriers between minima compared to CENP-A + CENP-C^{CM}.

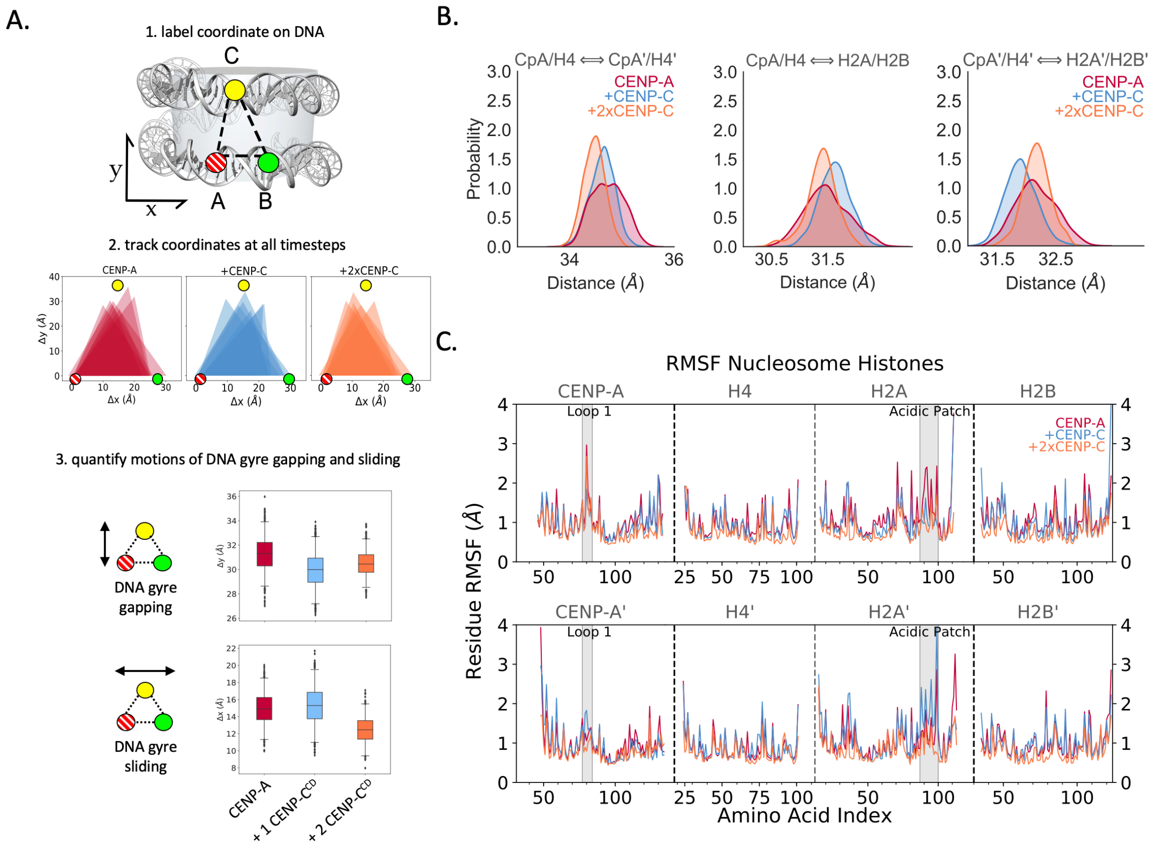

Figure S2 – Two CENP-C^{CM} fragment strengthens stiffening of CENP-A nucleosomes

(*A*) All-atom computational modeling of DNA gyre gapping or DNA gyre sliding of CENP-A nucleosome alone or bound to either 1 or 2 CENP-C^{CM} fragments. (*B*) The distance between the center of mass (COM) of histone dimers is shown in red for CENP-A, blue for CENP-A + 1 CENP-C^{CM}, and in orang for CENP-A + 2 CENP-C^{CD}. Two CENP-C^{CM} fragment exaggerated the COM distances compared to a single CENP-C^{CM} fragment, which means that 2 CENP-C^{CM} further induces a global loss of CENP-A nucleosome flexibility. (*C*) Residue root mean square fluctuations (RMSF) shows freezing of local flexibility in the CENP-A nucleosome shown in red, 1 CENP-C^{CM} bound shown in blue, and 2 CENP-C^{CD} bound shown in orange. In the region of CENP-C^{CM} binding, the first heterotypic half on the top panel, CENP-C is seen to freeze the acidic patch and the loop 1 region of CENP-A. One CENP-C^{CM} creates asymmetry, especially at the C-terminal end of H2A and H2B, this is abrogated when the second CENP-C^{CM} is bound. Dashed lines separate individual histones.

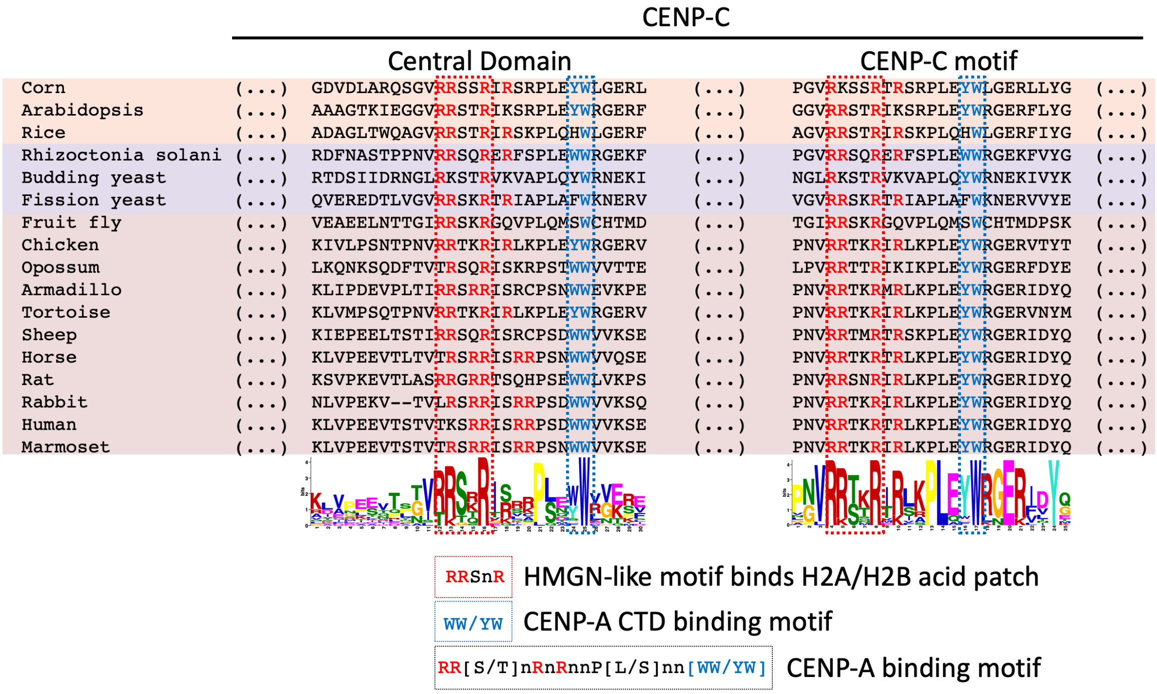

Figure S3 – CENP-C^{CM} and CENP-C^{CD} have conserved CENP-A nucleosome binding motifs

Alignment of various plant, fungal, and animal species shows that within the poorly conserved central domain and the well conserved CENP-C motif the RR(S/T)nR motif and the WW/YW motif are highly conserved. These two motifs are separated by 7 residues, creating a conserved H2A/H2B acid patch and C-terminal tail of CENP-A binding motif (RR(S/T)nRnRnnP(L/S)nn(WW/YW).

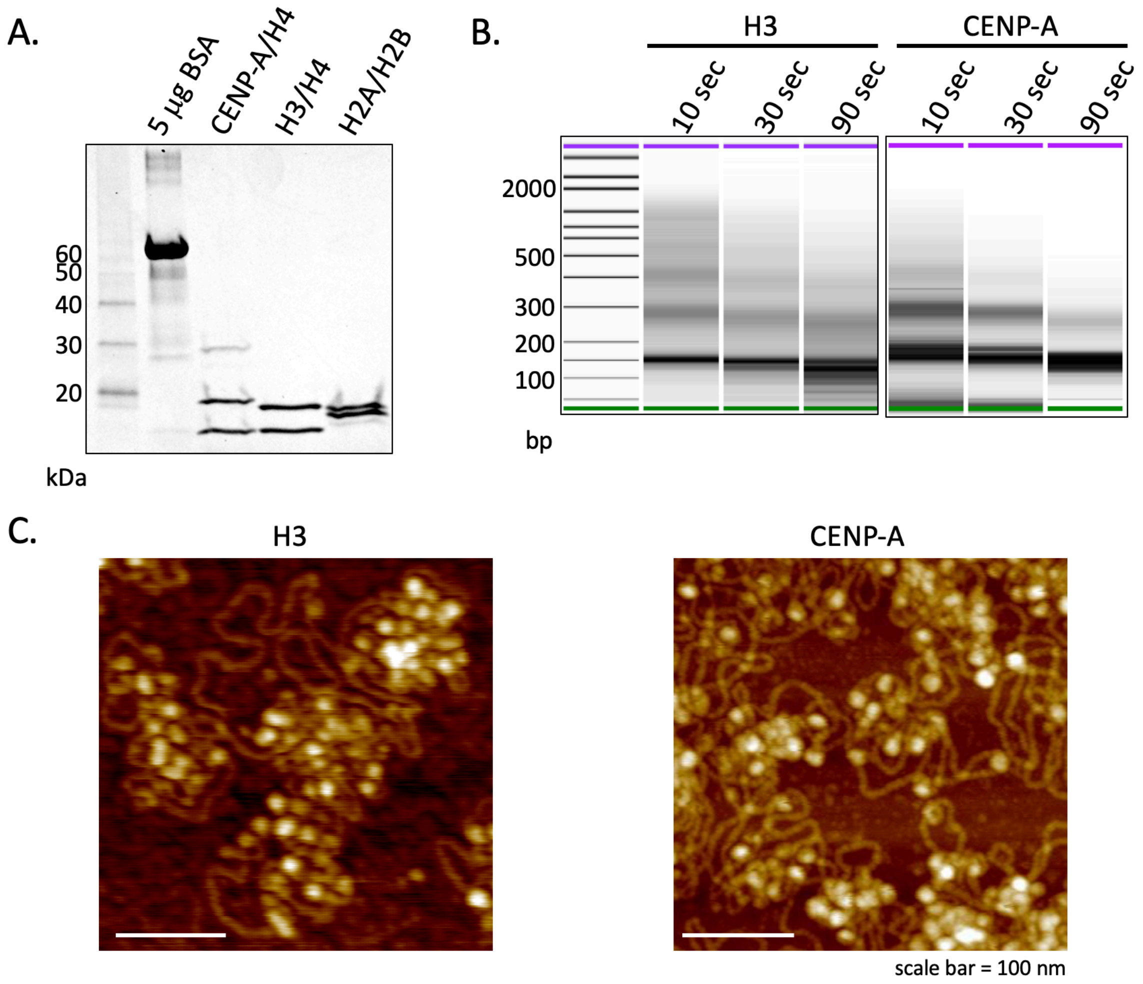

Figure S4 – Nucleosomal arrays show MNase ladder
(*A*) Histone protein concentration was determined by Coomassie staining. (*B*) BioAnalyzer results from 10, 30, and 90 seconds of MNase digested reconstituted H3 and CENP-A nucleosomes on 3 kbp plasmids a classic chromatin ladder. (*C*) Representative in air AFM images of H3 and CENP-A reconstituted chromatin.

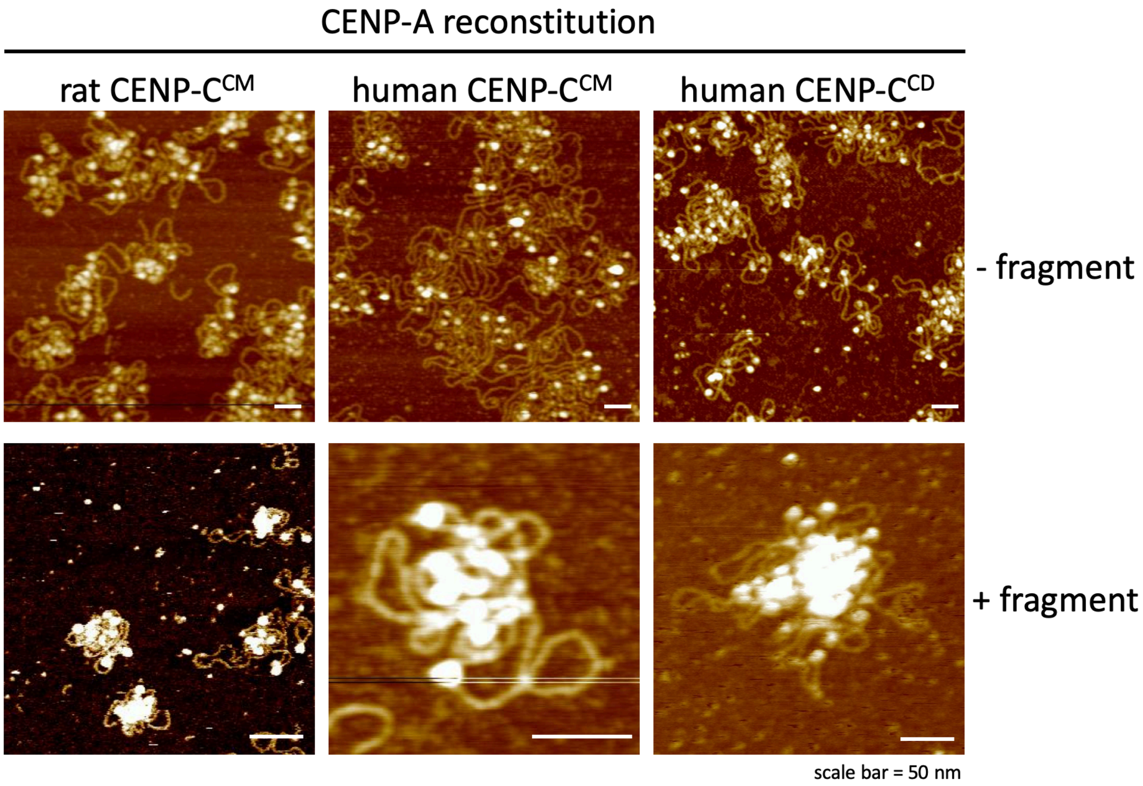

Figure S5 – CENP-C induces CENP-A chromatin clustering
Reconstituted CENP-A chromatin was incubated with either rat CENP-C^{CM}, human CENP-C^{CM}, or human CENP-C^{CD} fragments for 30 minutes. Cluster formation was observed with all three fragments.

A.

	H3	CENP-A	+ 1 CENP-C^{CM}	+ 2 CENP-C^{CM}
Predicted height (nm)	5.5	5.8	6.4	6.6
Predicted diameter (nm)	10.9	10.8	10.7	10.6

B.

	H3	CENP-A	+ 2x CENP-C^{CD}	+ 4x CENP-C^{CD}
Measured height (nm)	3.8±0.3	3.7±0.3	4.1±0.5	4.1±0.6
Measured diameter (nm)	14.0±1.2	13.7±1.0	13.5±0.9	14.0±1.2
Measured volume (nm^3)	393±68	370±61	394±61	426±61

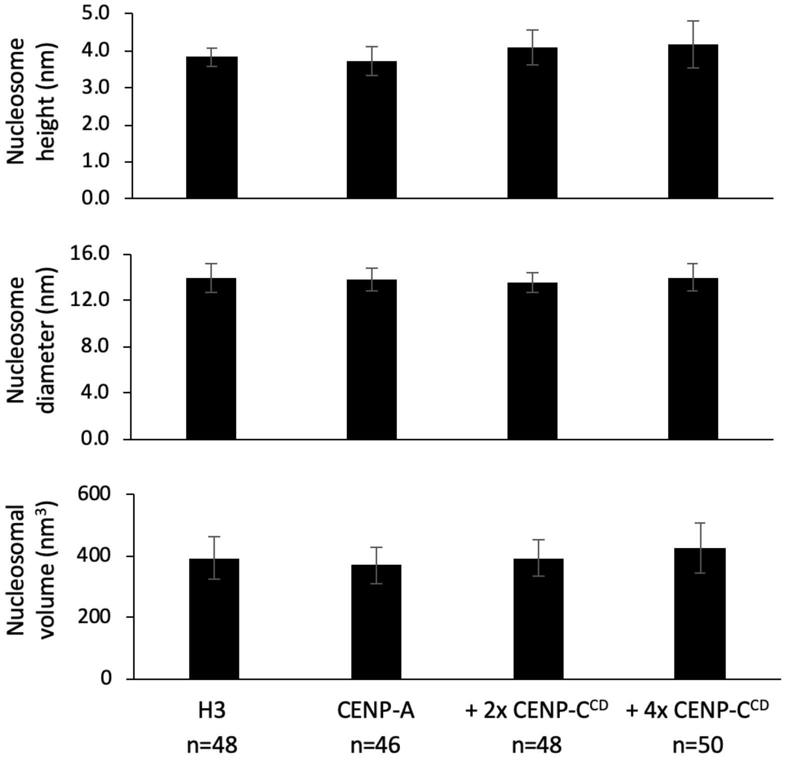

Figure S6 – CENP-C^{CD} modestly increases CENP-A nucleosome heights
(*A*) Height and diameter predictions from the computational modeling experiment described in Figure 1A. (*B*) CENP-C^{CD} modestly increases height of *in vitro* reconstituted CENP-A nucleosomes H3 and CENP-A nucleosome were in vitro reconstituted, and by in fluid AFM, we measured their dimensions (height, diameter, and volume). The height distribution is shown in the violin plot containing a bar plot. CENP-A nucleosomes are ever so slightly smaller than H3 nucleosomes. The addition of CENP-C^{CD} fragment, which can only bind CENP-A nucleosomes, we observed an increase in height and in a dose-dependent manner its volume.

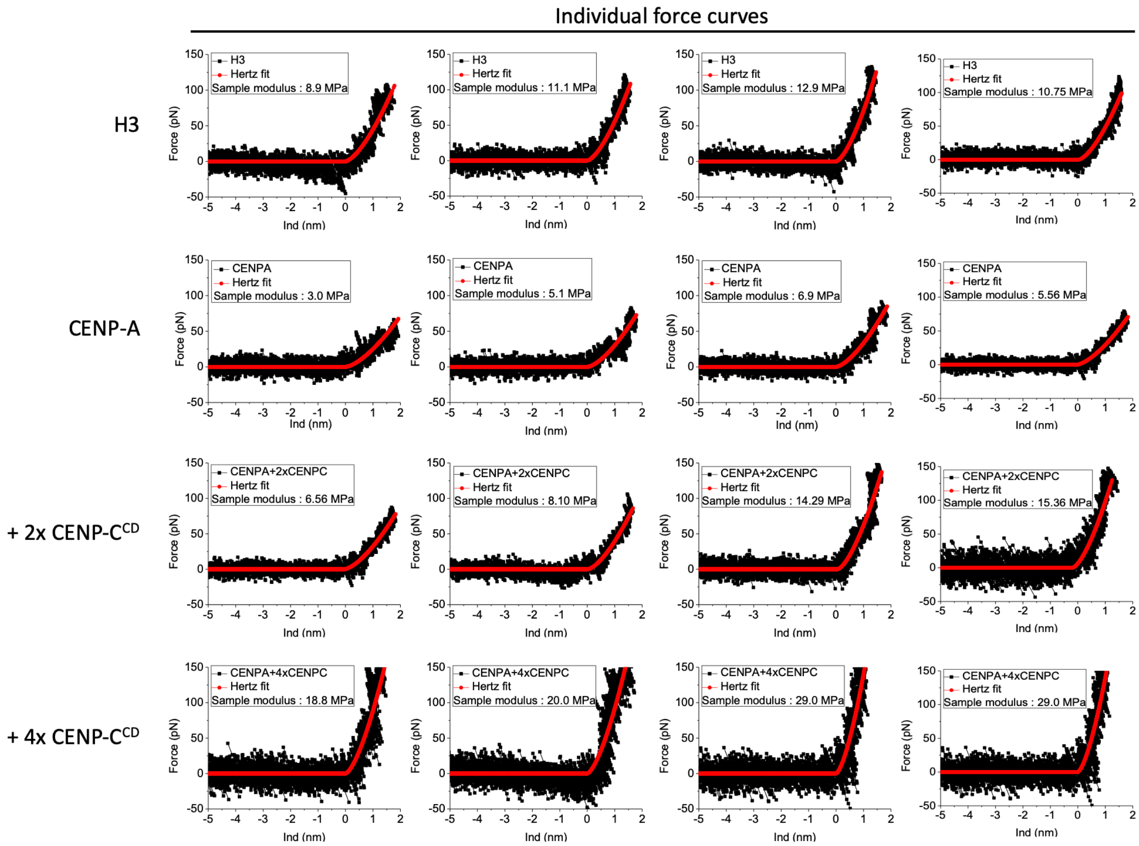

Figure S7 – Examples of force curve measurements

Four representative force curves for H3 nucleosomes, CENP-A nucleosomes, CENP-A nucleosomes with 2-fold excess CENP-C^{CD} fragments, and CENP-A nucleosomes with 4-fold excess CENP-C^{CD} fragments are shown.

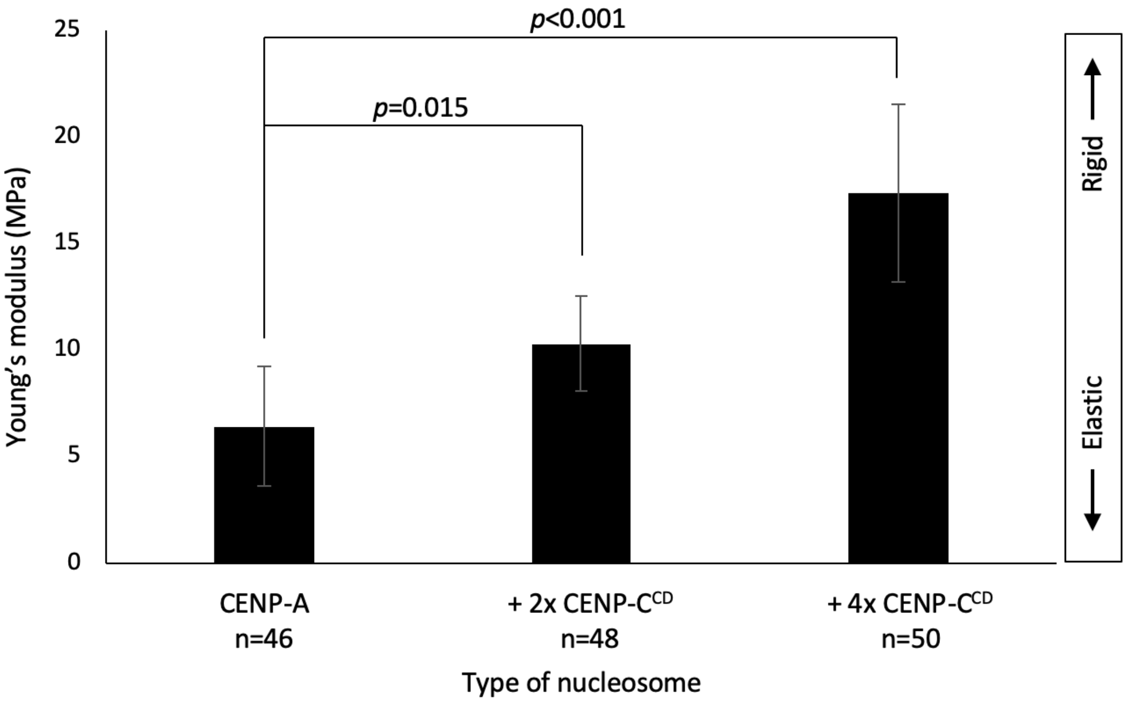

Figure S8 – 4-fold excess of CENP-C^{CD} results in further increased CENP-A nucleosomes rigidification.

Bar plot summarizing the Young's modulus values showing that CENP-A nucleosomes become stiffer upon addition of 2-fold excess CENP-C^{CD} (two-sided t-test p=0.015), and even stiffer upon addition of 4-fold excess CENP-C^{CD} binding (two-sided t-test p<0.001). ~1000 force curves were measured per condition.

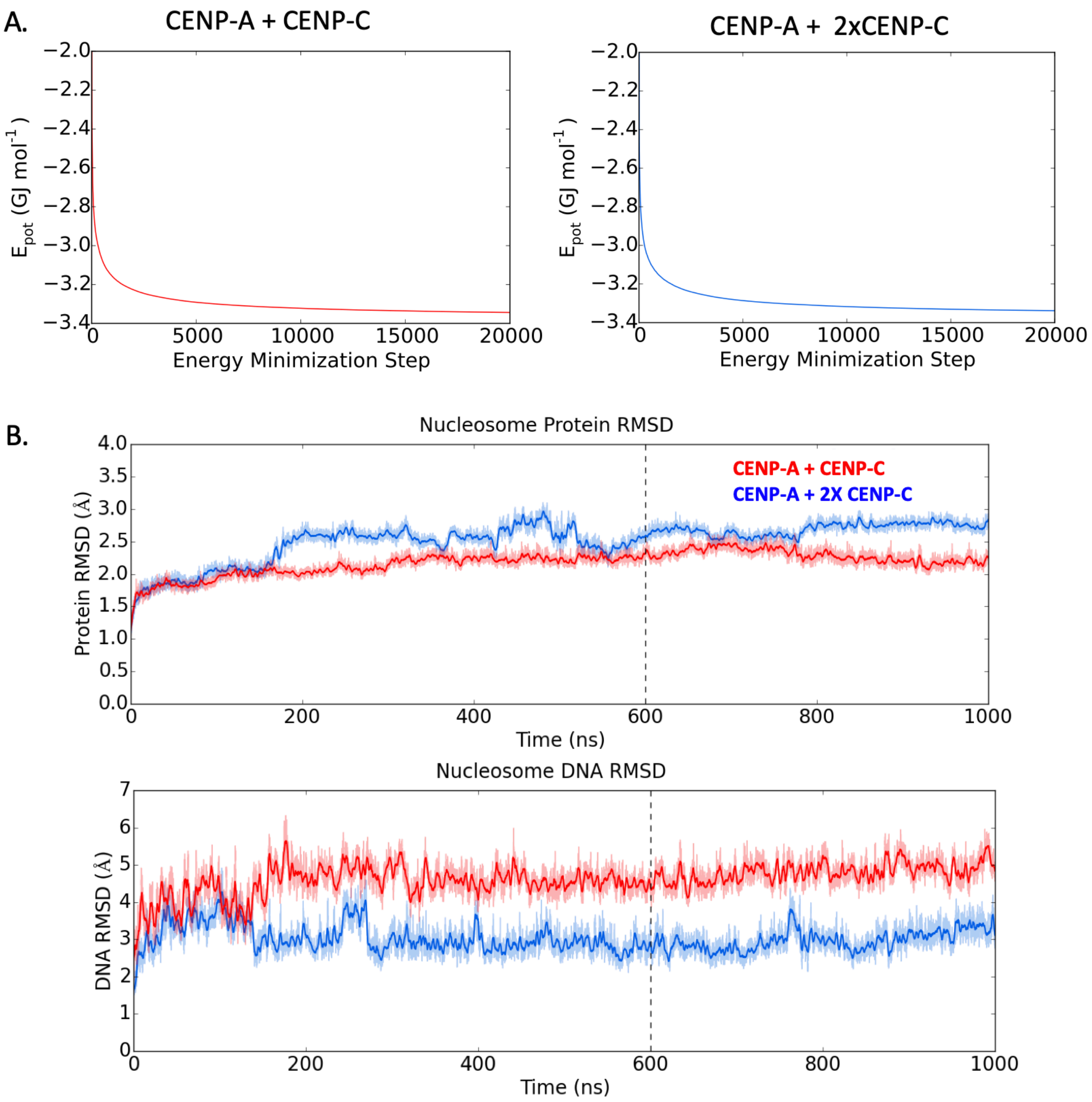

Figure S9 – Quality control of computational simulations

(*A*) The systems were energy minimized to allow for relaxation of potential clashes or energetically disfavorable rotamers and solvent or ion interactions. (*B*) The simulations ran for 1000 ns and then checked for equilibration by calculation of the root mean square displacement (RMSD) in comparison to the structure after minimization and equilibration. Data before 600 ns was cleaved from the analysis datasets.

Table S1. Quantification of nucleosomal dimensions by AFM analysis.
Data demonstrate that *in vitro* chromatin reconstitution yields equivalent dimensions for CENP-A and H3, but that CENP-A nucleosomes increase in height by ~0.4nm when bound to CENP-C^{CD}. Heights (nm), Diameters (nm),and volumes (nm^3) were calculated for representative particles of each class of nucleosome imaged by atomic force microscopy in-fluid conditions (*Methods*).

Supplemental Table S1: Quantification of nucleosome particles in H3, CENP-A, and CENP-A + CENP-C^{CD} conditions.

H3			CENP-A			2X CENP-C^{CD}			4X CENP-C^{CD}		
height(nm)	diameter (nm)	volume(nm3)	height(nm)	diameter (nm)	volume(nm3)	height(nm)	diameter (nm)	volume(nm3)	height(nm)	diameter (nm)	volume(nm3)
3.8	12.3	300.8	3.7	12.8	317.2	5.4	12.5	441.5	4.7	14.1	489.1
4.1	12.3	324.6	3.7	13.4	347.6	4.5	15.1	536.9	3.6	15.1	429.5
3.2	15.1	381.8	3.1	12.5	253.4	4.4	12.8	377.2	5.4	12.8	463.1
3.5	16.7	510.8	3.2	14.4	347.2	4.3	12.8	368.6	4.4	14.2	464.4
3.7	13.5	352.8	3.2	12.6	265.8	4.3	14.1	447.3	3.7	14.8	424.1
3.9	14.3	417.3	3.5	13.9	353.8	4.3	12.7	362.9	3.6	12.5	294.3
4.2	13.5	400.5	3.5	13.5	333.8	4.2	13.4	394.6	3.6	12.7	303.7
3.9	15.5	490.3	3.6	15	423.9	4.1	14.5	451.1	3.3	15.1	393.7
3.9	14.3	417.3	3.6	13.8	358.7	4	13.1	359.2	3.8	13.6	367.8
4	12.5	327.1	3.8	14.2	400.9	3.9	13.5	371.9	4.5	15.9	595.4
3.7	15.5	465.2	3.9	14	400.1	3.8	12.8	325.8	4.1	14.7	463.5
3.7	12.7	312.3	4.1	14.1	426.5	3.8	13.6	367.8	3.8	14.1	395.6
3.7	13.5	352.8	4.5	13.6	435.5	4.8	11.9	355.7	3.7	17.5	593.1
4	12.3	316.7	4.4	12.8	377.2	4.2	13.8	418.5	3.3	15.5	414.9
3.9	14.3	417.3	4.2	12.6	348.9	4.1	13.5	391.1	5.1	15.1	608.5
4	12.5	327.1	4	12.8	342.9	3.7	14.1	384.9	4.4	16.7	642.1
3.7	15.5	465.2	4	14.6	446.2	3.4	12.8	291.5	4.4	12.5	359.7
3.3	15.5	414.9	3.9	15	459.2	3.3	15.1	393.7	4.2	13.5	400.8
3.8	14.3	406.6	3.9	13.9	394.3	3.7	13.5	352.8	4.1	14.2	432.5
4.1	14.3	438.7	3.8	13.8	378.7	3.9	12.9	339.6	3.9	12.6	324.1
3.7	15.5	465.2	3.7	14.5	407.1	4.4	12.9	383.1	3.8	13.8	378.7
4.6	15.5	578.3	3.5	12.5	286.1	3.6	13.2	328.2	3.7	14.1	384.6
4	13.1	359.2	3.4	12.8	291.5	4.1	13.6	396.8	5.6	11.9	415.1
4.4	12.7	371.3	3.3	14.2	348.2	4.2	13.9	424.6	5.4	14.3	577.8
3.4	13.9	343.7	4.1	13.6	396.8	4.2	15	494.5	4.4	13.9	444.8
3.6	12.7	303.8	4.1	14.5	451.1	4.3	13.7	422.3	4.3	13.1	386.1
4.1	13.9	414.5	4	12.5	327.1	4.6	14.9	534.4	4.2	11.9	311.2
4.1	13.1	368.2	3.8	11.9	281.6	4.7	12.3	372.1	4.1	13.1	368.2
4.3	13.9	434.7	3.8	14.9	441.5	4.9	14.1	509.8	3.9	13.9	394.3
3.8	14.3	406.6	3.8	13.2	346.5	4.5	12.7	379.8	5.4	13.2	492.4
3.6	11.9	266.7	3.7	13.6	358.1	4.5	12.8	385.8	4.5	12.3	356.2
3.8	11.9	281.6	3.7	12.8	317.2	4.5	14.2	474.8	4.1	13.2	373.8
3.6	13.1	323.3	3.7	12.9	322.2	4.4	13.2	401.2	4.1	14.5	451.1
4.1	13.9	414.5	3.7	15.6	471.2	4.1	14.2	432.6	3.9	15	459.2
3.8	13.9	384.2	4.1	15.2	495.7	4.1	13.2	373.8	3.7	14.2	390.4
4.1	15.1	489.2	3.7	12.9	322.2	3.9	14.5	429.1	3.5	13.9	353.8
4.1	14.7	463.6	3.6	13.7	353.6	3.8	12.5	310.7	3.5	14.6	390.4
3.5	12.1	268.1	3.6	13.6	348.4	3.7	12.9	322.2	3.4	14.1	353.7
3.4	13.9	343.7	3.6	16.2	494.4	3.4	14.7	384.4	3.5	13.7	343.7
3.7	14.3	395.9	3.5	14.6	390.4	3.2	12.6	265.8	3.6	14.7	407.1
3.4	15.5	427.4	3.4	12.7	286.9	3.6	12.7	303.8	3.8	12.9	330.9

4.1	13.9	414.5	3.4	15.1	405.7	3.7	14.3	395.9	3.9	14.6	435.1
3.8	14.7	429.7	3.4	14.4	368.9	4.1	14.7	463.6	4.1	13.9	414.5
3.7	15.9	489.5				4.3	14.2	453.7	4.2	14.1	436.9
3.5	14.7	395.8				4.5	12.1	344.7	4.5	13.8	448.4
									4.9	14.6	546.6
									5.9	12.7	498.1

Table S2.Quantification of chromatin folding demonstrates that CENP-C increases CENP-A chromatin clustering.

Native chromatin incubated with or without the CENP-C fragment was visually inspected on AFM and identified as “open” or “clustered”. Total number of CENP-A clusters/total number of CENP-A nucleosome arrays per scan. Both analyses demonstrate that CENP-C increases CENP-A chromatin clustering (*Methods*).

	**ACA WT**			**ACA CENP-C^{CD}**		
	# arrays	# clusters	# clusters/# arrays	# arrays	# clusters	# clusters/# arrays
**Scan1**	14	5	0.36	19	7	0.37
**Scan2**	19	6	0.32	13	5	0.38
**Scan3**	22	6	0.27	14	6	0.43
**Scan4**	17	7	0.41	26	12	0.46
**Scan5**	34	13	0.38	15	6	0.40
**Scan6**	48	13	0.27	22	9	0.41
**Scan7**	40	16	0.40	24	11	0.46
	**ACA WT**			**ACA CENP-C^{CD}**		
	# arrays	# clusters	# clusters/# arrays	# arrays	# clusters	# clusters/# arrays
**Scan1**	31	10	0.32	38	28	0.74
**Scan2**	34	11	0.32	50	27	0.54
**Scan3**	26	14	0.54	49	35	0.71
**Scan4**	19	7	0.37	55	39	0.71
**Scan5**	28	8	0.29	43	21	0.49

Table S3. RNAP2 levels on centromeric chromatin are reduced under CENP-C over-expression conditions

Cells were transfected (or not) with full length CENP-C which was over-expressed (OE) for 3 days, native centromeric chromatin was extracted by CENP-C or ACA ChIP, from wildtype cells or CENP-C OE cells, in parallel. Chromatin was evaluated for RNAP2and CENP-A occupancy on Western blots. 3 independent replicates were quantified using the Licor scanner and automated software. Quantification of RNAP2 in CENP-C IP or ACA IP over Input demonstrates a suppression of RNAP2 levels on centromeric chromatin upon CENP-C OE, and a reduction of total CENP-A levels when RNAP2 is diminished.

Supplemental Table S3: Quantification of band intensity of RNA polymerase 2 and CENP-A western blots. Values are arbitrary units derived from LiCor's software (10^3).

**RNAP2**								
	WT					CENP-C^{OE}		
	Input	CENP-C	ACA			Input	CENP-C	ACA
Exp1	5.92	26.8	17.5		Exp1	6.52	9.12	8.76
Exp2	7.76	14.1	12.6		Exp2	9.36	7.5	10.8
Exp3	7.34	24.9	17.8		Exp3	6.31	8.07	7.67
		Ratio CpC/input	Ratio ACA/input				Ratio CpC/input	Ratio ACA/input
Exp1		4.53	2.96		Exp1		1.40	1.34
Exp2		1.82	1.62		Exp2		0.80	1.15
Exp3		3.39	2.43		Exp3		1.28	1.22
	mean	3.25	2.33			mean	1.16	1.24
	StDev	1.36	0.67			StDev	0.32	0.10
**CENP-A**								
	WT					CENP-C^{OE}		
	Input	CENP-C	ACA			Input	CENP-C	ACA
Exp1	0.98	8.63	13.2		Exp1	1.97	6.74	8.53
Exp2	0.57	10	11.4		Exp2	0.56	8.7	2.18
Exp3	0.38	1.71	2.23		Exp3	0.52	1.6	2.02
		Ratio CpC/input	Ratio ACA/input				Ratio CpC/input	Ratio ACA/input
Exp1		8.81	13.47		Exp1		3.42	4.33
Exp2		17.54	20.00		Exp2		15.54	3.89
Exp3		4.50	5.87		Exp3		3.08	3.88
	mean	10.28	13.11			mean	7.34	4.04
	StDev	6.65	7.07			StDev	7.10	0.25
